# Acid-Sensing Ion Channel 1/Calpain1 Activation Impedes Macrophage ATP-Binding Cassette Protein A1-Mediated Cholesterol Efflux Induced by Extracellular Acidification

**DOI:** 10.3389/fphys.2021.777386

**Published:** 2022-01-20

**Authors:** Yuan-Mei Wang, Mo-Ye Tan, Rong-Jie Zhang, Ming-Yue Qiu, You-Sheng Fu, Xue-Jiao Xie, Hong-Feng Gu

**Affiliations:** ^1^Hengyang Key Laboratory of Neurodegeneration and Cognitive Impairment and Institute of Neuroscience, Hengyang Medical College, University of South China, Hengyang, China; ^2^Department of Zhongjing Theory, College of Chinese Medicine, Hunan University of Chinese Medicine, Changsha, China; ^3^Hengyang Hospital of Traditional Chinese Medicine, Hengyang, China

**Keywords:** extracellular acidification, foam cells, ASIC1, calpain1, ABCA1, cholesterol efflux

## Abstract

**Background:**

Extracellular acidification is a common feature of atherosclerotic lesions, and such an acidic microenvironment impedes ATP-binding cassette transporter A1 (ABCA1)-mediated cholesterol efflux and promotes atherogenesis. However, the underlying mechanism is still unclear. Acid-sensing ion channel 1 (ASIC1) is a critical H^+^ receptor, which is responsible for the perception and transduction of extracellular acidification signals.

**Aim:**

In this study, we explored whether or how ASIC1 influences extracellular acidification-induced ABCA1-mediated cholesterol efflux from macrophage-derived foam cells.

**Methods:**

RAW 264.7 macrophages were cultured in an acidic medium (pH 6.5) to generate foam cells. Then the intracellular lipid deposition, cholesterol efflux, and ASIC1/calpain1/ABCA1 expressions were evaluated.

**Results:**

We showed that extracellular acidification enhanced ASIC1 expression and translocation, promoted calpain1 expression and lipid accumulation, and decreased ABCA1 protein expression as well as ABCA1-mediated cholesterol efflux. Of note, inhibiting ASIC1 activation with amiloride or Psalmotoxin 1 (PcTx-1) not only lowered calpain1 protein level and lipid accumulation but also enhanced ABCA1 protein levels and ABCA1-mediated cholesterol efflux of macrophages under extracellular acidification conditions. Furthermore, similar results were observed in macrophages treated with calpain1 inhibitor PD150606.

**Conclusion:**

Extracellular acidification declines cholesterol efflux *via* activating ASIC1 to promote calpain1-mediated ABCA1 degradation. Thus, ASIC1 may be a novel therapeutic target for atherosclerosis.

## Introduction

Acidic pH (pH < 7.0) of the intimal fluid in plaques has been regarded as a critical pathological feature of atherosclerosis (Morgan and Leake, 1993; Sneck et al., 2005). The extracellular acidic fluid is mainly resulted from local endometrial hypoxia in atherosclerotic lesions. As we know, under such a hypoxic condition, macrophages will shift their metabolism to anaerobic glycolysis, leading to excessive lactate production and proton extrusion (Zhang et al., 2019). Thus, the acidic intimal fluid is generated in atherosclerotic lesions (Back et al., 2019; Lee-Rueckert et al., 2020). In fact, acidification has been observed both in human and animal atherosclerotic lesions, especially in advanced plaques (Naghavi et al., 2002). Interestingly, extracellular acidification is often observed in the cultured macrophages when the cells are activated or exposed to oxidized low-density lipoprotein (ox-LDL). Recently, extracellular acidification has been confirmed to diminish macrophage cholesterol efflux and promote atherogenesis by unknown mechanisms (Lee-Rueckert et al., 2010). Therefore, clarifying the mechanisms by which extracellular acidification inhibits cholesterol efflux is critical to prevent macrophage-derived foam cells formation and atherosclerotic development.

Acid-sensing ion channel 1 (ASIC1), a member of the ENaC/degenerin family, is activated by elevated extracellular protons (Borg et al., 2020). This channel is widely expressed in nervous and vascular systems and has the unique property to be permeable to calcium (Bouron, 2020). Therefore, activation of ASIC1 enhances calcium influx and initiates intracellular signaling to trigger downstream events, such as inflammation and cell death (Wang and Xu, 2011; Zhang R. J. et al., 2020). ASIC1 plays crucial roles in physiological and pathophysiological processes, including vascular remodeling, cognitive function disorders, pancreatic cancer, and cerebral ischemic injury (Detweiler et al., 2019; Zhou et al., 2019; Zhu et al., 2021). Recently, Ni et al. (2018) reported that ASIC1 activation results in macrophage cell inflammatory response, which impedes ATP-binding cassette protein A1 (ABCA1)-mediated cholesterol efflux from the cells. However, whether the activation of ASIC1 directly influences macrophage cholesterol efflux and foam cell formation is still unclear.

Acid-sensing ion channel 1 is highly expressed in murine macrophages (Ni et al., 2018). Its activation increases intracellular calcium levels, which activates calcium-dependent protease. Calpain1, a calcium-sensitive cysteine protease, has been closely associated with ABCA1-cholesterol efflux (Hanouna et al., 2020). ABCA1 prevents atherogenesis *via* facilitating cholesterol efflux to lipid-free apolipoprotein A1 (ApoA1) from foam cells (Attie, 2007). However, ABCA1 is unstable and prone to be degraded by calpain1, leading to a decrease in ABCA1 expression on the cell surface and a subsequent reduction in cholesterol efflux (Yokoyama et al., 2012). Of note, extracellular acidification obviously decreased ABCA1 protein expression in macrophages and had no significant influence on ABCA1 mRNA levels (Lee-Rueckert et al., 2010, 2020). The discordances between ABCA1 protein and mRNA expression suggest that extracellular acidification decreases ABCA1 protein levels in macrophages *via* promoting its degradation. Given the activated ASIC1 has high permeability for calcium and elevated intracellular calcium increases calpain1 activity (Xiong et al., 2004; Stankowska et al., 2018), we speculate that extracellular acidification results in ASIC1/calpain1 pathway activation and subsequent ABCA1 protein degradation, thereby reducing ABCA1-mediated cholesterol efflux from macrophages.

This study investigated whether ASIC1 activation mediated intracellular lipid deposition and ABCA1 expression and explored the underlying mechanisms in macrophages challenged with acidic culture medium. Our results indicate that extracellular acidification promotes ASIC1 activation and lipid accumulation and decreases ABCA1 protein levels. Mechanistically, ASIC1 activation enhances calpain1 activity and subsequent increases in intracellular lipid deposition through impeding calpain1-mediated ABCA1 cholesterol efflux. Taken together, this study reveals that ASIC1 is critical in linking extracellular acidification to cholesterol efflux and maybe a novel target for atherosclerosis therapy.

## Materials and Methods

### Materials

Oxidized low-density lipoprotein (Ox-LDL) was obtained from Yiyuan Biotechnology (Guangzhou, China). Oil red O (ORO) and RPMI 1640 medium were obtained from Sigma-Aldrich (United States). Recombinant human ApoA1 and CCK-8 kit were provided by Beyotime Biotechnology (Shanghai, China). NBD-cholesterol was purchased from Thermo Fisher Scientific (United States). Anti-ASIC1 antibody was purchased from Alonome (State of Israel). Anti-calpain1 antibody and β-actin antibody were purchased from Proteintech (United States). Anti-ABCA1 antibody was purchased from Abcam (United Kingdom). Goat anti-rabbit antibody was bought from Cell Signaling Technology (United States). Alexa Fluor 488 Goat anti-rabbit antibody was from Jackson TmmunoResearch (United States). DAPI was obtained from Solarbio (Beijing, China). 1,1′-Dioctadecyl-3,3,3′,3′-tetramethylindocarbocyanine perchlorate (Dil) was bought from YEASEN Biotech Co., Ltd. (Shanghai, China). Amiloride was purchased from Sigma-Aldrich (United States), PD150606 was from Abcam Company (United Kingdom). PcTx-1 was acquired from MedChemExpress (United States).

### Cell Culture and Treatment

RAW 264.7 macrophages were obtained from the Institute of Cardiovascular Disease, University of South China. To generate foam cells, RAW 264.7 macrophages were incubated in a culture medium of pH 7.4, pH 7.0, or pH 6.5 with 25 μg/ml ox-LDL for 24 h. To sustain a stable extracellular pH, the macrophages were incubated in a CO_2_-independent medium (Invitrogen, United States) containing 10% fetal bovine serum (FBS, United States) and 4 mM L-glutamine at 37°C during the experiment (Xu et al., 2011). For pharmacological treatment, the macrophages were cocultured in ASIC inhibitor amiloride, calpain1 inhibitor PD150606, and ASIC1 inhibitor PcTx1 for 24 h, respectively.

### Cell Viability Assay

CCK8 kit (Beyotime Biotechnology, Shanghai, China) was used to detect cell viability and proliferation. Raw 264.7 macrophages were cultured in different pH media supplied with 25 μg/ml ox-LDL in a 96−well plate. After 24 h of treatment, CCK8 (10 μl per well) was incubated with cells for another 4 h at 37°C. Subsequently, the absorbance value of cultured cells was measured using a microplate reader at a wavelength of 450 nm.

### Oil Red O Staining

Oil red O staining assay was performed to measure the lipid accumulation in RAW 264.7 macrophages. Those cells were cultured in different pH media supplemented with 25 μg/ml ox-LDL in 6-well plates for 24 h. Then the cultured cells were harvested and fixed using 4% paraformaldehyde for 30 min at room temperature. After being washed three times with phosphate-buffered saline (PBS), the cells were stained with ORO for 15 min. Subsequently, hematoxylin was used to counterstain those samples for 15 s. Lipid accumulation was evaluated using a microscope (Thermo Fisher Scientific, China) and quantified using Image pro plus 7.0 software (Media Cybernetics, Inc., United States).

### Western Blot Assay

Cells were collected for protein extraction as described previously (Gu et al., 2017). In brief, after being washed with ice-cold PBS, cell pellets were lysed in RIPA buffer supplemented with protease and phosphatase inhibitors. Protein content was measured using a BCA protein assay kit (Beijing ComWin Biotech, China). Equal amounts of protein samples (10 μg) were loaded on 10% SDS-PAGE for separation. Then, those separated proteins were transferred to polyvinylidene fluoride membranes (Millipore, United States) and blocked with 5% bovine serum albumin for 1 h. Subsequently, the membranes were incubated with primary antibodies against ASIC1 (1:1,000), ABCA1 (1:1,000), calpain1 (1:1,000), and β-actin (1:2,000) overnight at 4°C. After being washed 3 times, the membranes were incubated with second antibodies conjugated with horseradish peroxidase (HRP). Finally, protein bands were visualized using an enhanced chemiluminescence detection system. Image J software was used to quantify the immunoblots.

### Real-Time Quantitative PCR

RAW 264.7 macrophages were used to extract total RNA using a TRIzol reagent (Invitrogen) following instructions. The pure and concentrated RNA was then used to synthesize complementary DNA using a cDNA reverse transcription kit (Applied Biosystems, United States). The sequence of ABCA1 primers were 5′-ATGCCAATAACCCTTGCTTCCG -3′ and 5′-ATGTCCCTAATGCTGGTGTC CTT -3′. ABCA1 mRNA level was analyzed using ABI PRISM 7900 sequence detection system (Applied Biosystems).

### Detection of Triglycerides and Total Cholesterol Contents

The contents of triglycerides and cholesterol in RAW 264.7 macrophages were determined using a commercially available quantitation kit (Nanjing Jiancheng Bioengineering institute, China). Briefly, RAW 264.7 macrophages were treated with an FBS-free medium. After 12 h, macrophages were cultured in different pH media with or without amiloride (0, 50, and 100 μM). Then, the content of cellular cholesterol and triglycerides was measured following the instructions of the manufacturer.

### Immunofluorescent Staining Assay

RAW 264.7 macrophages were stained by immunofluorescence to detect the co-localization of ASIC1 and cellular membrane. RAW 264.7 cells were cultured in different pH media in 6-well plates for 24 h. Then, DiI (1:200) was used to treat cells for 20 min. After being washed three times, the cells were fixed with 4% paraformaldehyde for 30 min at room temperature. Afterward, RAW 264.7 cells were preincubated with 5% goat serum to avoid non-specific antibody binding. Then, the cells were incubated with anti-ASIC1 antibody (1:500) overnight at 4°C. The immune complexes were visualized using Alexa Fluor 488-labeled secondary antibody. DAPI staining was used to indicate the nuclei. Images were obtained using a fluorescence microscope (Thermo Fisher Scientific, China).

### ATP-Binding Cassette Protein A1-Mediated Cholesterol Efflux Assay

RAW 264.7 macrophages were incubated with different pH media containing 25 μg/ml ox-LDL in 6-cell plates (1 × 10^5^/cell) for 24 h. Then, the cells were incubated with 5 μmol/L NBD-cholesterol in the phenol red-free RPMI 1640 medium for 4 h to be loaded with cholesterol. After being washed 3 times with PBS, cell layers were incubated in the absence or presence of the indicated concentrations of drugs for an additional 4 h. Finally, cholesterol efflux proceeded for 4 h in a medium containing 20 μg/ml ApoA-1. The fluorescence intensity of the culture medium and cell lysate was determined using a microplate spectrophotometer. The efflux rate was measured by the ratio of medium fluorescence counts to total fluorescence intensity (medium counts + cells lysate counts) × 100%.

### Statistical Analysis

All data were shown as mean ± SEM. The Student’s *t*-test was used to analyze means between two groups. Differences among the groups were analyzed by one-way ANOVA using SPSS 20 software (International Business Machines Corporation, United States). Statistical significance was considered when *P* < 0.05.

## Results

### Extracellular Acidification Increases Intracellular Lipid Accumulation

**FIGURE 1:**
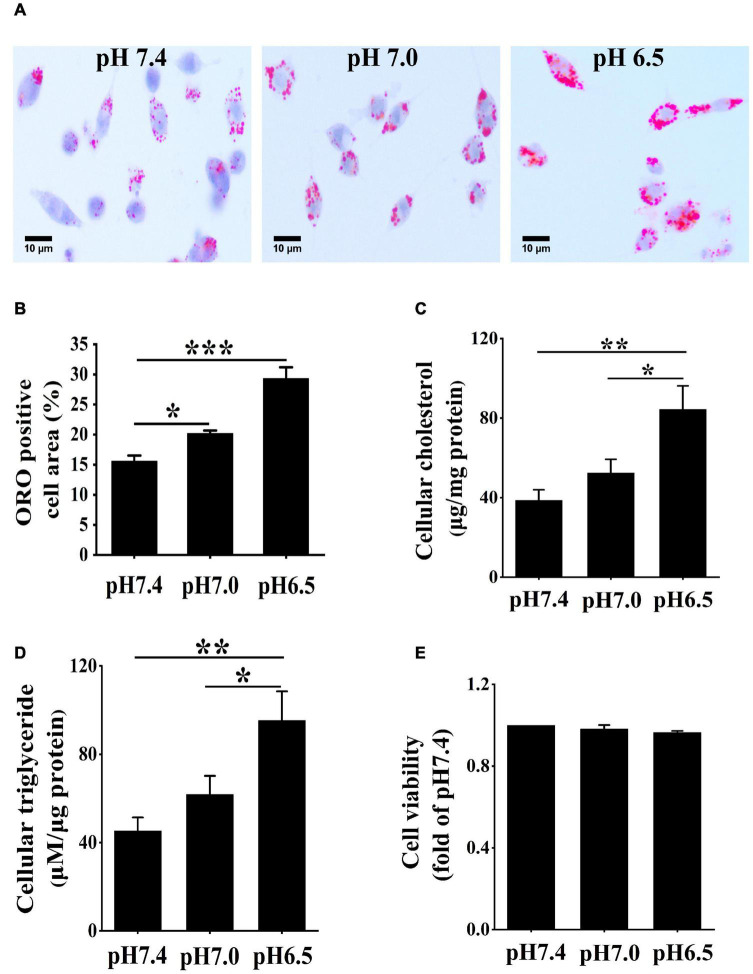
Extracellular acidification aggregates lipid deposition in macrophages. **(A)** RAW 264.7 cells were cultured in 25 μg/ml oxidized low-density lipoprotein (ox-LDL) in different pH media for 24 h. Lipid accumulation was detected by Oil red O (ORO) staining. **(B)** ORO staining positive areas were quantified. **(C,D)** Intracellular cholesterol and triglycerides contents were determined by enzymatic assay. **(E)** CCK-8 kit was used to detect cell viability and proliferation. Results are expressed as the mean ± SEM. Statistical analysis was performed by one-way analysis of variance (ANOVA). *P < 0.05; **P < 0.01; ***P < 0.001.

To acquire the role of extracellular acidification in macrophage-derived foam cells, RAW 264.7 macrophage cells were cultured in 25 μg/ml ox-LDL and different acidic pH media for 24 h. ORO staining was used to detect lipid accumulated in the cells under different pH media. Compared with pH 7.4 medium, lipid accumulation was significantly increased in the pH 7.0 group and pH 6.5 group (Figures 1A,B). Furthermore, the contents of triglycerides and cholesterol were measured in different pH media (Figures 1C,D). The results demonstrated that triglycerides and cholesterol contents were both significantly increased in the pH 7.0 and pH 6.5 groups as compared with the pH 7.4 group. In addition, the results of the CCK-8 assay revealed that pH 7.0 and pH 6.5 culture media had less influence on cell viability (Figure 1E). These results demonstrate that extracellular acidification directly promotes lipid accumulation in RAW 264.7 cells.

### Extracellular Acidification Promotes the Expressions and Translocation of Acid-Sensing Ion Channel 1 in RAW 264.7 Cells

**FIGURE 2:**
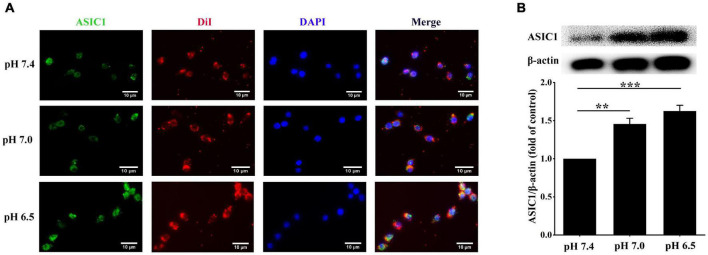
Extracellular acidification promotes acid-sensing ion channel 1 (ASIC1) expression and translocation in macrophages. RAW 264.7 macrophages were incubated with ox-LDL (25 μg/ml) in different pH media for 24 h. **(A)** Representative immunofluorescence of ASIC1 and DiI in RAW 264.7 macrophages. The scale bar is 10 μm. **(B)** The membrane protein levels of ASIC1 were measured using Western blot (WB) analysis. Data were shown as the mean ± SEM from 3 to 4 independent experiments. Statistical analysis was performed by one-way ANOVA. **P < 0.01, ***P < 0.001.

Next, we explored the potential signaling that could be responsible for the extracellular acidification-induced lipid deposition in RAW 264.7 cells. Activation of ASIC1 contributes to neuronal cell death in the contest of tissue acidosis (Borg et al., 2020). We clarified whether similar mechanisms are implicated in increasing lipid accumulation in RAW 264.7 cells. We examined the expression and membrane translocation of ASIC1 in RAW 264.7 cells using immunofluorescent staining and Western blotting, respectively. As indicated in Figure 2A, DiI perchlorate (a far-red fluorescent to track cell membrane) was used to stain cellular membranes. The co-localization (indicated by yellow color) of ASIC1 and DiI was much higher in the pH 7.0 and 6.5 groups than those in the pH 7.4 group, respectively. Western blotting results also show that ASIC1 protein expression in the cell plasma membrane of these two acidic pH groups was markedly increased as compared with the pH 7.4 group, respectively (Figure 2B). These results suggest that ASIC1 may be associated with lipid accumulation in RAW 264.7 cells induced by extracellular acidification.

### Extracellular Acidification Promotes Calpain1 Expression *via* Acid-Sensing Ion Channel 1 Activation

**FIGURE 3:**
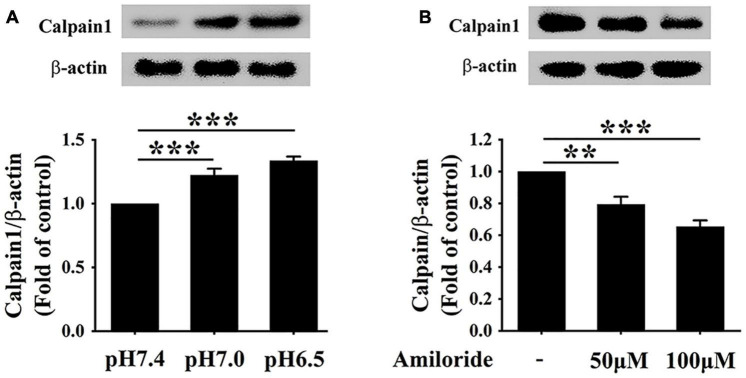
Extracellular acidification enhances calpain1 expression *via* activating ASIC1 in macrophages. **(A)** RAW 264.7 cells were incubated in different pH media with ox-LDL for 24 h, and then the expression of calpain1 was determined using WB analysis. **(B)** RAW 264.7 macrophages were incubated in pH 6.5 medium containing 25 μg/ml ox-LDL with amiloride (0, 50, and 100 μM) for 24 h. Calpain1 expression was determined using WB analysis. Data were shown as the mean ± SEM from 3 to 4 independent experiments. Statistical analysis was performed by one-way ANOVA. **P < 0.01; ***P < 0.001.

Considerable evidence proves that the ASIC1 activation enhances calcium influx, which results in the activation of calpain1 (Verheijden et al., 2018; Wang et al., 2020). Therefore, we investigated the relationship between ASIC1 and calpain1 expression in RAW 264.7 cells under the extracellular acidification condition. As expected, consistent with the increases in ASIC1 expressions, calpain1 protein levels were significantly elevated in the pH 7.0 group and pH 6.5 group as compared with the control group (Figure 3A). Of note, treatment with ASIC1 inhibitor amiloride abolished the increases in calpain1 expression under extracellular acidification conditions (Figure 3B). These results illustrate that ASIC1 activation enhances calpain1 expression in RAW 264.7 cells induced by extracellular acidification. Collectively, these results imply that ASIC1/calpain1 activation may involve extracellular acidification-promoted lipid deposition in RAW 264.7 cells.

### Extracellular Acidification Inhibits ATP-Binding Cassette Protein A1 Expression and ATP-Binding Cassette Protein A1-Mediated Cholesterol Efflux

**FIGURE 4:**
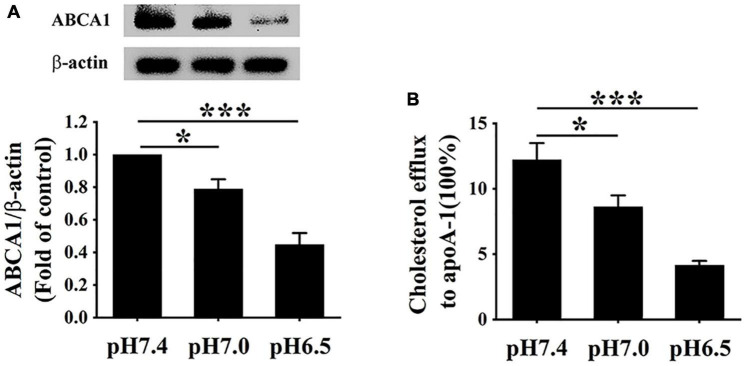
Extracellular acidification decreases ABCA1 protein levels and cholesterol efflux of macrophages. **(A)** RAW 264.7 cells were incubated in different pH media with 25 μg/ml ox-LDL for 24 h. The protein levels of ABCA1 were valued using WB analysis. **(B)** ABCA1-mediated cholesterol efflux was analyzed using an NBD-cholesterol kit. Data were shown as the mean ± SEM from 3 to 4 independent experiments. Statistical analysis was performed by one-way ANOVA. *P < 0.05; ***P < 0.001.

ATP-binding cassette transporter A1 protein on the surface of macrophages can easily be degraded by calpain1-mediated proteolysis (Martinez et al., 2003). Given the elevated levels of calpain1 in RAW 264.7 cells, we hypothesized that extracellular acidification might decrease the protein levels of ABCA1. The ABCA1 protein levels were detected using Western blotting, and ABCA1-mediated cholesterol efflux was measured using a high-throughput NBD-labeled cholesterol efflux assay. As expected, Western blotting results indicated that the levels of ABCA1 were much lower at pH 6.5 than at pH 7.0 and pH 7.4, respectively (Figure 4A), revealing that extracellular acidification decreased the ABCA1 protein expression in RAW 264.7 cells. Consistent with the lowered expression of ABCA1, cholesterol efflux was markedly reduced in the pH 6.5 group as compared with that in the pH 7.4 group and pH 7.0 group (Figure 4B). Altogether, these results demonstrate that extracellular acidification impedes ABCA1-mediated cholesterol efflux, thereby accelerating lipid accumulation in RAW 264.7 cells.

### Extracellular Acidification Increases Lipid Accumulation and Decreases ATP-Binding Cassette Protein A1-Mediated Cholesterol Efflux *via* Acid-Sensing Ion Channel 1 Activation

**FIGURE 5:**
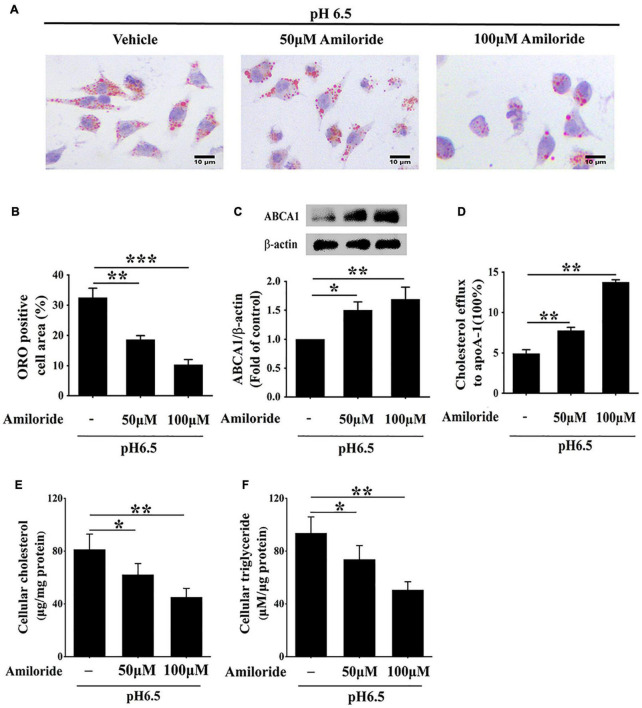
Inhibition of ASIC1 impedes lipid accumulation and promotes ABCA1-mediated cholesterol efflux of macrophages. RAW 264.7 macrophages were cultured in 25 μg/ml ox-LDL and amiloride (0, 50, and 100 μM) under pH 6.5 condition for 24 h. **(A)** Lipid deposition was detected by ORO staining. **(B)** ORO positive areas were quantified. **(C)** ABCA1 protein levels were measured using WB analysis. **(D)** ABCA1-mediated cholesterol efflux was determined using an NBD-cholesterol kit. **(E,F)** Intracellular cholesterol and triglycerides contents were determined by enzymatic assay. Data were shown as the mean ± SEM from 3 to 4 independent experiments. Statistical analysis was performed by one-way ANOVA. *P < 0.05; **P < 0.01; ***P < 0.001.

Given that ASIC1 expression is increased in RAW 264.7 macrophages challenged with extracellular acidification, we determined the effect of this receptor on lipid accumulation and ABCA1-dependent efflux of the cells. RAW 264.7 cells were exposed to an acidic culture medium (pH 6.5) with or without ASIC1 inhibitor amiloride for 24 h. Lipid deposition, ABCA1 expression, and cholesterol efflux were measured (Figures 5A–D). ORO results showed that lipid accumulation (indicated by ORO positive area) was obviously attenuated in groups treated with 50 and 100 μM amiloride as compared with the control group (Figures 5A,B), respectively, indicating that ASIC1 activation promotes lipid accumulation induced by acidic pH.

Cholesterol efflux prevents lipid accumulation in macrophage cells. Hence, we further investigated whether inhibition of ASIC1 could reverse the decreases in ABCA1 expression and ABCA1-dependent cholesterol efflux of RAW 264.7 cells under extracellular acidification conditions. Western blotting results showed that ABCA1 protein levels of ASIC1 inhibitor groups were significantly increased as compared with that of the control group (Figure 5C). Consistent with the changes in ABCA1 expression, the percentage of cholesterol efflux to ApoA1 was much higher in the ASIC1 inhibitor groups than that of the control group (Figure 5D). Taken together, these results demonstrate that extracellular acidification suppresses ABCA1-mediated cholesterol efflux *via* ASIC1 activation, thereby promoting macrophage lipid accumulation.

Moreover, the storage levels of triglycerides and cholesterol were detected in RAW 264.7 macrophages cultured in a pH 6.5 medium with or without amiloride for 24 h (Figures 5E,F). The contents of triglycerides and cholesterol were significantly decreased in 50 and 100 μM amiloride groups compared with those of the control group. These results indicate that ASIC1 activation promotes lipid accumulation.

### Extracellular Acidification Promotes Lipid Accumulation and Inhibits ATP-Binding Cassette Protein A1-Mediated Cholesterol Efflux *via* Acid-Sensing Ion Channel 1/Calpain1 Pathway

**FIGURE 6:**
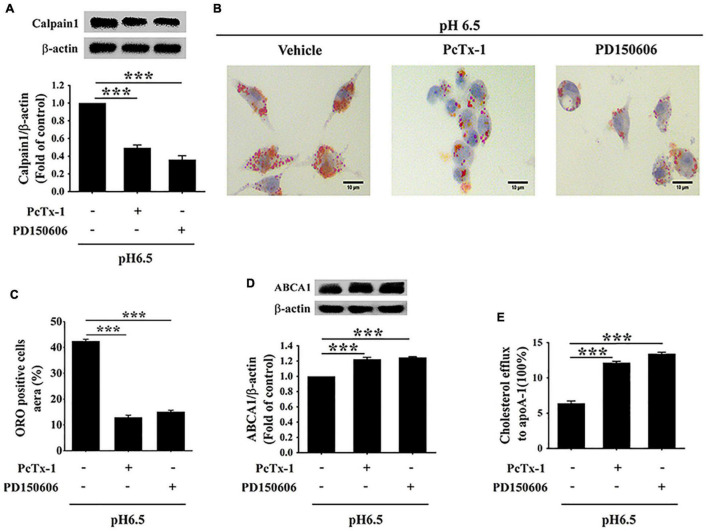
Extracellular acidification increases macrophage lipid deposition *via* activating ASIC1/calpain1 to reduce ABCA1-dependent cholesterol efflux. RAW 264.7 macrophage cells were incubated in pH 6.5 culture medium with or without ASIC1 specific inhibitor PcTx-1 (100 ng/ml) or calpain1 inhibitor PD150606 (50 μM) for 24 h. **(A)** Calpain1 expression was evaluated using WB analysis. **(B)** Lipid deposition was detected using ORO staining. **(C)** ORO staining positive areas were quantified. **(D)** ABCA1 expression was measured using WB analysis. **(E)** ABCA1-mediated cholesterol efflux was measured using the NBD-cholesterol kit. Data were shown as the mean ± SEM from 3 to 4 independent experiments. Statistical analysis was performed by one-way ANOVA. ***P < 0.001.

To further verify whether extracellular acidification increased lipid deposition and decreased cholesterol efflux *via* the activation of ASIC1/calpain1 signaling, RAW 264.7 cells were treated with PcTx-1 (a specific ASIC1 inhibitor) or PD150606 (a specific calpain1 inhibitor). We found that ASIC1 inhibitor PcTx-1 abolished the increases in calpain1 expression and lipid accumulation in RAW 264.7 cells under an acidic microenvironment (Figures 6A–C). Moreover, PcTx-1 treatment reversed the influence of extracellular acidification on the inhibition of ABCA1 protein expression and cholesterol efflux (Figures 6D,E). Interestingly, similar results were obtained when the cells were treated with calpain1 inhibitor PD150606 (Figures 6D,E). Furthermore, real-time PCR (RT-PCR) results indicated that there was no significant difference in the ABCA1 mRNA expression both in PcTx-1 and calpain1 groups as compared with the pH 6.5 group (Supplementary Figure 1), suggesting that these two inhibitors had less influence on the ABCA1 mRNA expression. Taken together, these data manifest that the activation of ASIC1/calpain1 signaling contributes to extracellular acidification-promoted lipid accumulation in RAW 264.7 cells.

## Discussion

Acid-sensing ion channel 1 has been confirmed to play critical roles in pathophysiological acidosis occurring during neurological disorders, such as ischemic stroke and various neurodegeneration (Friese et al., 2007; Borg et al., 2020). However, the potential roles of ASIC1 in macrophage lipid metabolism under extracellular acidification conditions are unclear. This study shows that an ASIC1-dependent increase in calpain1 activity and reduction in ABCA1 protein levels are evoked in RAW 264.7 cells cultured in an acidic medium. Importantly, inhibiting the activation of the ASIC1/calpain1 pathway by the inhibitors PcTx-1 and PD150606 not only restored ABCA1 protein levels but also significantly facilitated ABCA1-mediated cholesterol efflux and diminished lipid deposition in RAW 264.7 cells exposed to the extracellular acidification. These findings present a novel mechanism that extracellular acidification diminishes ABCA1-dependent cholesterol efflux and ultimately induces foam cell formation *via* activation of the ASIC1/calpain1 pathway.

Acidic intimal fluid pH prevails in local areas of atherosclerotic plaques, where macrophage-derived foam cells locate (Liu et al., 2019). Furthermore, macrophages exposed to modified LDL are capable of generating acidic pericellular environments with pH values even lower than 5.0 (Naghavi et al., 2002; Sluimer et al., 2008). In this study, we first built an extracellular acidification-induced macrophage foam cell model by culturing RAW 264.7 cells in pH 6.5 medium, which may closely mimic the acidic microenvironment-surrounded macrophages within atherosclerotic lesions. Our results confirmed that extracellular acidification notably diminished ABCA1-mediated cholesterol efflux and exacerbated lipid deposition in RAW 264.7 macrophages. Consistently, the acidic extracellular pH also profoundly compromised ABCA1-dependent cholesterol efflux from human monocyte-derived macrophage foam cells (Lee-Rueckert et al., 2020). To clarify the mechanisms underlying extracellular acidification-induced lipid accumulation in RAW 264.7 macrophage cells, we explored the ASIC1/calpain1/ABCA1 signaling in which the acidic extracellular pH involved, including changes in ASIC1 expression and cell membrane translocation, calpain1 and ABCA1 protein levels, and ABCA1-mediated cholesterol efflux.

In this study, we showed the critical role of ASIC1 in extracellular acidification-induced lipid accumulation in RAW 264.7 macrophage cells. ASIC1 is widely expressed in the nervous and cardiovascular systems (Arun et al., 2013; Qiang et al., 2018). Under normal conditions, this protein is primarily expressed in the nucleus. Once exposed to an extracellular acidic pH value, ASIC1 will translocate from nucleus to cell membrane (Zhang Y. et al., 2020), thereby being activated by extracellular protons to trigger downstream signaling cascades such as calpain1 activation and RIP1 interaction (Wang et al., 2015; Stankowska et al., 2018). To explore whether this channel is involved in extracellular acidification-induced lipid accumulation, we first clarified the changes in ASIC1 expression in the membrane when RAW 264.7 cells were cultured in an acidic medium. Our results indicated that ASIC1 is expressed in RAW 264.7 cells and that extracellular acidification significantly promoted its expression and cell membrane translocation. Consistent with the increase in ASIC1 expression in the membrane, intracellular lipid deposition aggregated obviously. Notably, ASIC1-specific inhibitor PcTx-1 treatment significantly attenuated lipid accumulation in the cells under extracellular acidification conditions. These findings reveal that extracellular acidification promoted lipid accumulation in RAW 264.7 cells *via* ASIC1 activation.

ATP-binding cassette transporter A1 impedes foam cell formation and atherogenesis by facilitating cholesterol efflux (Phillips, 2018). Several studies indicate that ABCA1-mediated cholesterol efflux is decreased both in cultured macrophage cells and in atherosclerotic plaques, and this decreased capability is closely related to extracellular acidic pH (Yu et al., 2013; Jin et al., 2018). However, the mechanism underlying extracellular acidification-induced reductions in cholesterol efflux is unknown. Thus, in our study, we explored the influences of ASIC1 activation on the ABCA1 expression in RAW 264.7 cells under extracellular acidification conditions. Our results present that the ABCA1 protein level is decreased under such conditions, coupled with the diminished capability of cholesterol efflux in RAW 264.7 cells. As expected, the decreases both in ABCA1 protein levels and cholesterol efflux were reversed by treatment with ASIC1 inhibitors amiloride and PcTx-1. Taken together, these findings demonstrate that ASIC1 activation by extracellular acidification lowers ABCA1 protein levels, thereby decreasing cholesterol efflux from RAW 264.7 macrophages.

The mechanism of extracellular acidification-induced ABCA1 protein degradation results from the activation of the ASIC1/calpain1 pathway. Several lines of evidence have identified that ASIC1 activation enhances calcium influx (Gonzalez Bosc et al., 2016). Calpain1 is a calcium-dependent cysteine protease and its activation promotes the degradation of ABCA1 protein (Martinez et al., 2003; Yokoyama et al., 2012). Therefore, we assumed that extracellular acidification enhanced the activity of calpain1 *via* ASIC1 activation. In fact, the activation of ASIC1 significantly increases the expression of calpain1 in RAW 264.7 macrophages cultured in an acidic medium, indicating that ASIC1/calpain1 pathway is activated in this context. To clarify the role of this signaling activation in extracellular acidification-induced changes in ABCA1 protein levels and cholesterol efflux, we employed specific inhibitors to inhibit ASIC1/calpain1 activation. We found that ASIC1 inhibitor treatments remarkably lowered the levels of calpain1 in RAW 264.7 cells exposed to the acidic culture medium. Furthermore, our results confirmed that calpain1 inhibitor PD150606 treatment profoundly elevated ABCA1 protein levels of the cells under extracellular acidification conditions. Accordingly, ABCA1-dependent cholesterol efflux is increased, and lipid accumulation was reduced. Notably, there was no significant difference in ABCA1 mRNA levels between the PcTx-1 group, the PD150606 group, and the pH 6.5 alone group, indicating that activation of ASIC1/calpain1 had no significant influence on the macrophage ABCA1 mRNA expression under acidic conditions. Collectively, these results revealed that the activation of ASIC1 by extracellular acidification increases the calpain1 activity, thereby facilitating calpain1-mediated ABCA1 degradation.

It is undeniable that there are certain limitations in this work. First, in this study, we explored the effect of the ASIC1/calpain1 pathway in extracellular acidification-induced macrophage lipid accumulation only using pharmacological inhibitors and not using siRNA to validate our findings. Second, the pilot study revealed the role of ASIC1/calpain1 activation in macrophage foam cell formation *in vitro*, and its actual atherogenic effect needs to be further verified *in vivo* through ASIC1 knockout and siRNA. Nonetheless, the results provided new insights into the correlation between extracellular acidification and ABCA1-mediated cholesterol efflux impairment, with ASIC1 acting as a link.

## Conclusion

Extracellular acidification promotes ASIC1 activation and ABCA1 degradation *via* enhancing calpain1 activity, leading to decreased ABCA1 protein levels and diminished cholesterol efflux in macrophages, ultimately causing lipid deposition, and macrophage-derived foam cell formation. Inhibition of ASIC1/calpain1 signaling to restore ABCA1-mediated cholesterol efflux may be a promising therapeutic approach for atherosclerotic diseases.

## Data Availability Statement

The original contributions presented in the study are included in the article/Supplementary Material, further inquiries can be directed to the corresponding author/s.

## Author Contributions

Y-MW performed writing the original draft, conception, experimental execution, and investigation. M-YT contributed to data curation, experimental execution, and verification. R-JZ contributed to data curation and experimental execution. M-YQ contributed to investigation. Y-SF performed writing the original draft. X-JX contributed to verification and resources. H-FG contributed to project administration and resources, and edited the draft. All authors contributed to the article and approved the submitted version.

## Conflict of Interest

The authors declare that the research was conducted in the absence of any commercial or financial relationships that could be construed as a potential conflict of interest.

## Publisher’s Note

All claims expressed in this article are solely those of the authors and do not necessarily represent those of their affiliated organizations, or those of the publisher, the editors and the reviewers. Any product that may be evaluated in this article, or claim that may be made by its manufacturer, is not guaranteed or endorsed by the publisher.

## Supplementary Material

Supplementary Figure 1 ASIC1/calpain1 has no effect on the ABCA1 mRNA level. RAW 264.7 macrophage cells were cultured in pH 6.5 medium in the absence or presence of ASIC1 specific inhibitor PcTx-1 (100 ng/ml) or calpain1 inhibitor PD150606 (50 μM) for 24 h. The mRNA level of ABCA1 was measured using real-time PCR (RT-PCR). Data were shown as the mean ± SEM from 3 to 4 independent experiments. Statistical analysis was performed by one-way ANOVA.
