# Supplementary figures and images for: Acid-Sensing Ion Channel 1/Calpain1 Activation Impedes Macrophage ATP-Binding Cassette Protein A1-Mediated Cholesterol Efflux Induced by Extracellular Acidification
Source: Front Physiol. 2022 Jan 20;12:777386. doi: 10.3389/fphys.2021.777386 (PMC8811198; doi:10.3389/fphys.2021.777386)

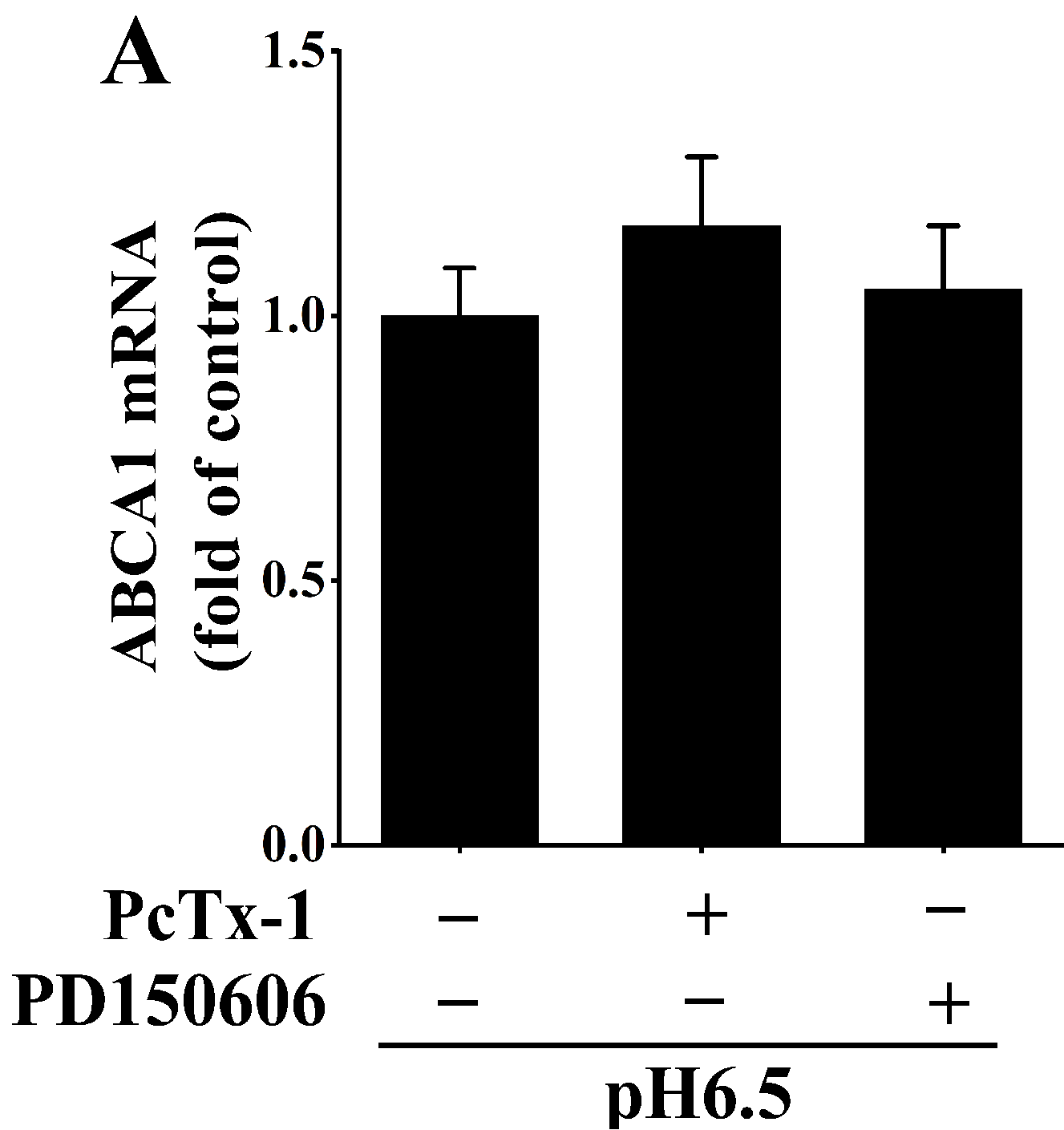

Supplement: Supplementary Figure 1 — ASIC1/calpain1 has no effect on the ABCA1 mRNA level. RAW 264.7 macrophage cells were cultured in pH 6.5 medium in the absence or presence of ASIC1 specific inhibitor PcTx-1 (100 ng/ml) or calpain1 inhibitor PD150606 (50 μM) for 24 h. The mRNA level of ABCA1 was measured using real-time PCR (RT-PCR). Data were shown as the mean ± SEM from 3 to 4 independent experiments. Statistical analysis was performed by one-way ANOVA. [file Image_1.TIF]
